# COVID-19 vaccine preferences for pregnant and lactating women in Bangladesh and Kenya: a qualitative study

**DOI:** 10.3389/fpubh.2024.1412878

**Published:** 2024-08-14

**Authors:** Jessica L. Schue, Berhaun Fesshaye, Emily Miller, Prachi Singh, Rupali J. Limaye

**Affiliations:** ^1^Department of International Health, Johns Hopkins Bloomberg School of Public Health, Baltimore, MD, United States; ^2^Department of Epidemiology, Johns Hopkins University, Bloomberg School of Public Health, Baltimore, MD, United States; ^3^Department of Health, Behavior, and Society, Johns Hopkins University, Bloomberg School of Public Health, Baltimore, MD, United States

**Keywords:** vaccine safety, COVID-19, Bangladesh, Kenya, pregnant women, lactating women

## Abstract

COVID-19 was responsible for more than 7 million deaths globally, as well as numerous morbidities and social and economic effects. While COVID-19 vaccines were seen as a marvel of science by the scientific community, much of the public had concerns related to COVID-19 vaccines, with certain groups—such as pregnant and lactating women—having specific concerns related to vaccine effects on their pregnancy and breast milk. In this qualitative study, we interviewed stakeholders in Bangladesh (*n* = 26) and Kenya (*n* = 94) who affect the decision-making process related to COVID-19 vaccine acceptance among pregnant and lactating women. These included pregnant and lactating women themselves, community gatekeepers or family members, healthcare workers, and policymakers. Several themes related to confidence and vaccine preference emerged. Stakeholders indicated a lack of confidence related to non-mRNA vaccines due to safety concerns, number of doses, and media coverage; lack of confidence related to mRNA vaccines due to safety concerns; and preference for non-mRNA vaccines due to health system compatibility and availability. While COVID-19 vaccine availability in much of the world—particularly in low-and middle-income countries—affected the public’s ability to have a choice in the vaccine they received, there were evident vaccine preferences. As the public health world will continue to face other infectious disease outbreaks, bolstering vaccine confidence broadly and specifically related to new technologies will be paramount to realize the individual-and population-level benefits of life-saving vaccines.

## 1 Introduction

As of April 2024, the COVID-19 pandemic has been responsible for over 7 million deaths worldwide (1). The pandemic has also significantly altered the vaccine landscape, as it spurred cooperation to facilitate new vaccine technologies and regulatory approvals at a pace not previously seen (2). While this expedited timeline was seen as a miracle of science for public health broadly and vaccine scientists specifically, the general public expressed concerns about the speed at which COVID-19 vaccines were made available and the perceived newness of the mRNA technology (3). These concerns were exacerbated due to the presence of an infodemic alongside the pandemic, with the ubiquity of vaccine misinformation further contributing to an erosion of trust in public health institutions (4).

These COVID-era challenges spotlighted the urgent need to build trust in public perceptions of vaccines, particularly newer vaccines. Compared to pediatric vaccine acceptance, adult vaccine acceptance differs widely, and coverage is suboptimal (5). In addition, particular adult populations—including pregnant and lactating women—have discrete and specific concerns that have not been adequately addressed affecting their vaccine acceptance, even though they are at higher risk for vaccine-preventable disease complications (6). While COVID-19 vaccination is widely recommended for pregnant and lactating women at present, this was not the case earlier in the pandemic, and there has been and continues to be variability in policy recommendations across countries (7). Global data indicate that while COVID-19 vaccines are recognized as generally safe and effective for mothers and their babies, maternal vaccination is an underutilized public health mechanism for mitigating the effects of vaccine-preventable disease (8).

The first approved COVID-19 vaccine, a nucleoside-modified messenger RNA (mRNA) vaccine developed by BioNTech and Pfizer, received emergency use authorization in the United Kingdom on 2 December 2020 (9). The World Health Organization approved the vaccine for emergency use a few weeks later on 31 December 2020 (10). In Bangladesh, the COVID-19 vaccination rollout began in January 2021 with the purchase of 700,000 doses of the Oxford-AstraZeneca (OAZ) vaccine, with the first mRNA vaccines being available in June 2021 (11–13). As of October 2021, pregnant women in Bangladesh were allowed to receive COVID-19 vaccines during pregnancy with qualifications; lactating women were permitted to receive COVID-19 with no qualifications (14). Kenya received its first batch of COVID-19 vaccines, 1.02 million doses of the OAZ vaccine, on 6 March 2021; mRNA vaccines were not available until September 2021 (15, 16). Pregnant and lactating women in Kenya were eligible for COVID-19 vaccination after a risk/benefit consultation with their provider starting in August 2021; in December 2021, this policy was revised to remove the provider consultation requirement (17). Globally, there was stark inequity of vaccine access, with higher-income countries (HICs) hoarding over half of the global supply, their collective doses outnumbering the quantity needed (18). In 2021, HICs had ordered over 70% of five available COVID-19 vaccines, despite comprising only 16% of the global population (3). Since then, HICs have received billions of surplus doses, in contrast with many low-and middle-income countries having inadequate dose numbers for their populations (19). Furthermore, cold chain requirements for OAZ vaccine products were more conducive to the structural systems present in low-and middle-income countries than the newer mRNA technology (20). Given this, perceptions regarding vaccine brand preferences were a critical factor in vaccine confidence.

Given that the COVID-19 vaccines were new vaccines, there was explicit attention given to the effects of the vaccines. Reports of adverse effects from two mRNA vaccines, (21) including the death of 23 older adult patients after receiving an mRNA vaccine (22), likely led to skepticism about the largely unknown technology during the early days of the vaccine rollout. However, studies conducted in the United States and Poland found participants preferred mRNA vaccines over other types (23, 24). A study in the Philippines found that vaccine brand hesitancy was common among adults, with less reported acceptance toward Sinovac-CoronaVac and mRNA vaccines (25). While COVID-19 vaccine preferences existed in many settings globally, supply constraints and inequitable vaccine distribution hindered these preferences. Evidence of global vaccine brand inequity was highlighted as reports of the OAZ COVID-19 vaccine’s possible association with blood clots led high-income countries, such as Denmark and Australia, to limit or completely discontinue its use (26). However, Pacific island countries and areas had access to only OAZ vaccines and were unable to adjust their policies and use (26).

During the pandemic, COVID-19 vaccine availability and recommendations differed by country, particularly as related to pregnant and lactating women. We explored the decision-making process among Bangladeshi and Kenyan pregnant and lactating women and other relevant stakeholders related to COVID-19 vaccines. Given that decision-making does not occur in a vacuum, we were interested in understanding the decision-making process among pregnant and lactating women themselves, as well as those that influenced their vaccine decision-making process. We did not explicitly ask about vaccine preferences related to mRNA or non-mRNA vaccines; however, it is within this larger study that preferences related to COVID-19 vaccines emerged. In this study, we seek to summarize COVID-19 vaccine preferences and how they relate to vaccine confidence in Bangladesh and Kenya for pregnant and lactating women during the pandemic.

## 2 Methods

In Bangladesh, we interviewed 16 healthcare workers (eight who served rural communities and eight who served urban communities) and 10 policymakers from three different levels of the health system—national, divisional, and district—for a total of 26 interviews. Participants were recruited from the capital, Dhaka, and five different communities in the Rangpur Division in northern Bangladesh: Rangpur city (urban), Kanchibari (rural), Gaibandha (urban), Bamandanga (rural), and Ramjiban (rural). In Kenya, we conducted in-depth interviews with a diverse set of audiences that may influence the vaccine decision-making process of pregnant or lactating women: pregnant or lactating women (*n* = 29), male family members of pregnant or lactating women or community gatekeepers (*n* = 35), healthcare workers (*n* = 20), and policymakers (*n* = 10). Participants were recruited from three counties, with two communities in each county: Garissa (rural), Kakamega (rural and urban), and Nairobi (urban); see Table 1 for a list of sampled populations by country.

**Table 1:** Sampled populations across Kenya and Bangladesh.

	Bangladesh			Kenya			
	Rangpur division^1^		Dhaka	Garissa	Kakamega		Nairobi
Target population type	Urban	Rural	Urban	Rural	Urban	Rural	Urban
Pregnant and Lactating Women (PLW)	–	–	–	8	4	6	11
Community members (family members, religious leaders, community leaders)	–	–	–	8	2	10	15
Healthcare providers (HCPs) (midwives, nurses, doctors, immunizers)	7	9	–	6	4	4	6
Policymakers (divisional, district, and national levels)	5	–	5	2	2	–	6
Total	12	9	5	24	12	20	38

1 Includes districts of Rangpur, Ramjiban, Bamandanga, Kanchibari.

Data were collected in April–August 2022 in Bangladesh and August–September 2021 in Kenya. Interview instruments were pre-tested in both countries and included questions related to risk perception for the baby and the mother, vaccine efficacy, self-efficacy to get the vaccine, safety concerns, community norms, and vaccine experiences. Data collectors participated in a 3-day training exercise after completing an online human ethics training. Participants were recruited from various health clinics across the nine communities, and policymakers in both countries were identified through ministry contacts. If a participant met the inclusion criteria and agreed to participate, oral consent was obtained. Interviews were conducted in either English, Swahili, Bengali, or other local languages as necessary in a semi-private setting or via Zoom. All interviews were audio recorded. Members of the study team transcribed and translated the transcripts into English. All data were stored on encrypted servers, and only members of the study team had access to the data. Study activities involving in-person interaction, including training and data collection, were conducted following COVID-19 safety protocols per the Ministries of Health in both countries.

A team of seven used a grounded theory approach to analyze the data. The team conducted three rounds of open coding to develop, refine, and finalize a code list. Two members of the team conducted inter-rater reliability with ∼10% of the transcripts that neither of them had coded. Reliability was calculated by comparing coding compatibility on each of the transcripts chosen, and the average reliability score was >90%. The team then identified themes and sub-themes. Data were managed using ATLAS.ti. This study received ethics approval from the Johns Hopkins Bloomberg School of Public Health Institutional Review Board, the Bangladesh Medical Research Council, and the Scientific and Ethics Review unit with Kenya Medical Research Institute.

## 3 Results

**Figure 1:**
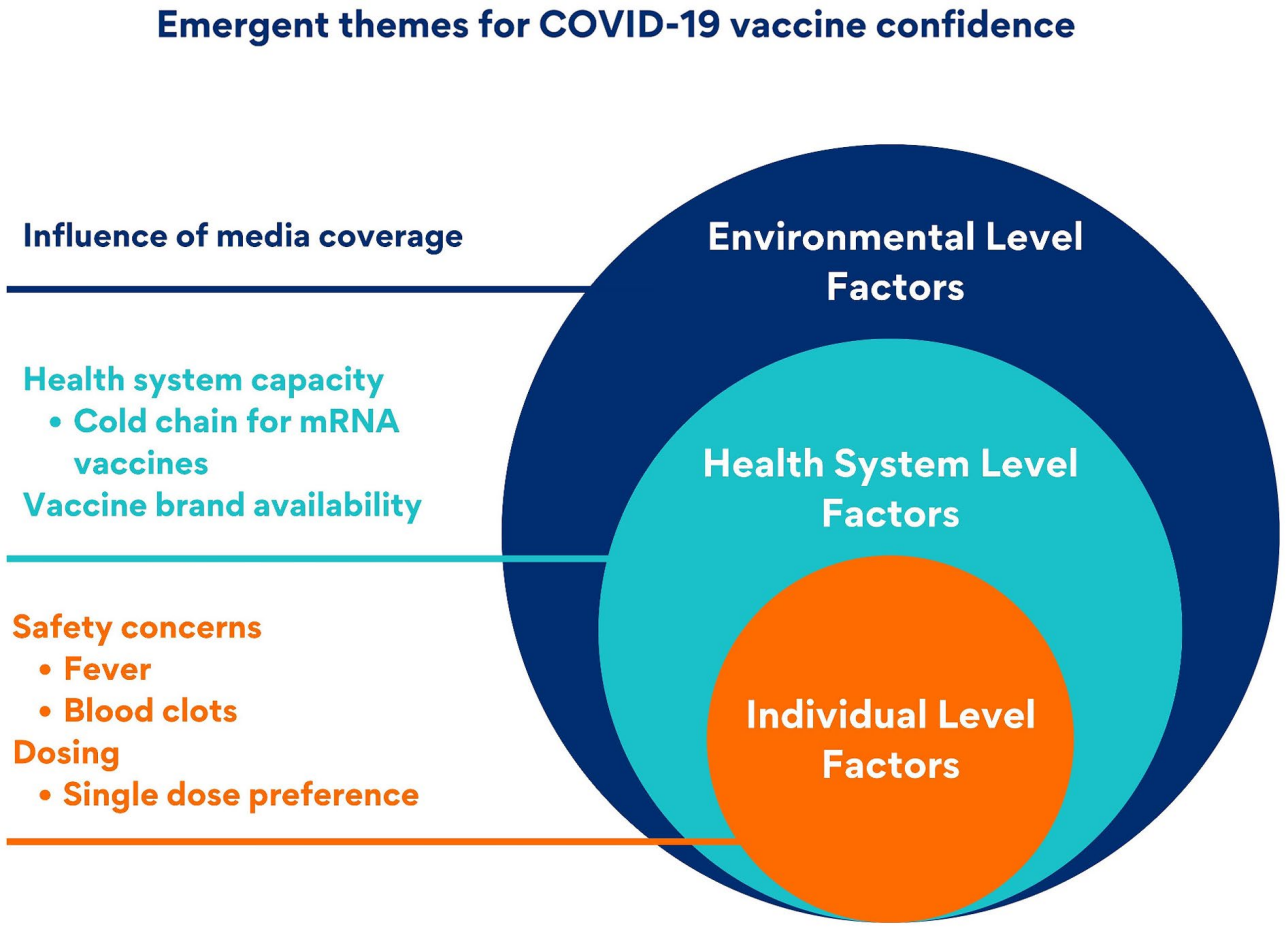
Multi-level factors affecting COVID-19 vaccine confidence: an adapted socio-ecological model.

Three key themes emerged related to COVID-19 vaccine confidence: (1) lack of confidence related to non-mRNA vaccines due to safety concerns, number of doses, and media coverage; (2) lack of confidence related to mRNA vaccines due to safety concerns; and (3) preference for non-mRNA vaccines due to health system compatibility and availability. The emergent themes were categorized into three levels using the socio-ecological model as a framework. Individual-level factors included perceived vaccine safety and dose number preferences. The health system level included cold chain requirements of mRNA vaccines and the availability of vaccine brands. The environmental level included influence from media reporting on vaccine safety (Figure 1).

### 3.1 Lack of confidence related to non-mRNA vaccines: safety concerns, number of doses, and media coverage

Stakeholders articulated reasons why communities were hesitant toward non-mRNA vaccines. These included safety concerns related to side effects (fever and blood clots), number of doses, and media coverage related to vaccine safety.

This Bangladeshi healthcare worker informed us that OAZ caused side effects such as fever and was thus not recommended for pregnant and breastfeeding women: “So first, we got the AstraZeneca. Because of AstraZeneca, you have high fever. Then we got the instruction not to give the vaccine to the pregnant and breastfeeding mothers, since a high fever was a side effect of AstraZeneca. Then the people did research and found out that it is okay to give this to pregnant mothers and breastfeeding mothers. But, if you give it to breastfeeding mothers, then maybe the breastfeeding baby might also get the fever (from the AstraZeneca vaccine). But, if the baby will eat something else, other than drinking mother’s milk, in those cases, we can give the COVID vaccine, but for 24 h, the baby cannot be breastfed. First, this was the instruction. Then after that, the instruction was 12 h, not 24 h. Then a vaccine came that was called Sinovac or Sinopharm, from China. That vaccine had very little side effect, almost none. So, for that, the instruction was that we can give it to the woman right after delivery, you can give it 2–3 months after delivery, and you can give it to pregnant mothers too since there are no side effects. This is what our supervisors explained to us. But before when we had given AstraZeneca, the instruction was that, since it had side effects, we are better not giving it to pregnant and breastfeeding mothers. Because of its side effects, we were told not to give AstraZeneca to pregnant and lactating women.” (*Male healthcare provider, rural, Bamandanga, Bangladesh*). In addition to concerns about getting a fever, this religious leader in Kenya referred to his community’s concerns related to blood clots linked to the OAZ vaccine: “Initially they (the community) had mixed reactions and actually most of them are waiting to see the reaction of those people who have been vaccinated. You could hear words from America that this particular vaccine has side effects like blood clots, but now they have faith, now that they have been assured by the government and we have not experienced any case within the community where one received vaccination and died or the person was crippled. And so, with this kind of assurance, and from what they have attested, they are able to say yes to the vaccine.” *(Community member 1, urban, Nairobi, Kenya).*

There were several instances related to preferences in dosing. When asked about her family’s vaccine intentions, this lactating woman from Kenya informed us that her family preferred the Johnson & Johnson vaccine over the OAZ vaccine: “They are planning to be vaccinated, but they do not want two jabs. They want Johnson & Johnson. My partner has not been vaccinated—he is waiting for Johnson & Johnson. That is what he said, he does not want to be injected twice with AstraZeneca.” *(Lactating woman, rural, Kakamega, Kenya).*

Finally, stakeholders informed us that the media played a role related to vaccine preferences. This Kenyan healthcare worker asserted that the media reported on safety concerns related to the OAZ vaccine: “There is also another issue—the media was reporting that COVID vaccines, especially the AstraZeneca vaccine, is not safe. This led to many questions: will the government take any action to look and see whether that information is true or not? And if that information is true, what action will the government take? Or are there other vaccines that can be substituted for this one? Or has the government put in place mechanisms or measures to know if this vaccine is safe for human uptake or not?” *(Male healthcare provider, rural, Garissa, Kenya).*

### 3.2 Lack of confidence related to mRNA vaccines: safety concerns

While the mRNA vaccines were eventually adopted for use in Bangladesh for pregnant and breastfeeding women, the government scrutinized the performance of the vaccine in the Bangladeshi population before rolling out a national campaign. This Bangladeshi policymaker asserted that while WHO recommended the Pfizer vaccine, additional observation was employed because it was an mRNA vaccine: “If the immunization committee of WHO gives recommendation, Bangladesh takes up the recommendation immediately. However, they do not suggest using it blindly with the recommendation. They (conduct a) trial for it. At first, they vaccinated 100–200 people. Then they observe those people for 7–10 days. So, after observing, (if there are no issues), approval of each vaccine was given. Pfizer is exceptional because it is used by so many people globally. However, Pfizer is an mRNA vaccine, so doctors suggested to spend time (examining its effect on people). It was given to probably 500 people who were then observed for a week. Then, the country did the national campaign.” *(National policymaker, urban, Dhaka, Bangladesh).*

Stakeholders informed us about concerns related to the safety of mRNA vaccines, including blood clots, and this healthcare worker from Kenya discussed the many myths in Kenya related to COVID vaccines, including the mRNA vaccines: “For COVID vaccines, we have so many myths, but when we really go and see the materials and the literature they give out, we have seen it is a very important vaccine. People are saying that (the mRNA vaccines) are going to give you blood clotting, but what I see is that (getting vaccinated) is very important.” *(Female healthcare provider 1, rural, Garissa, Kenya).* Similarly, another Kenyan healthcare worker alluded to hesitancy among other healthcare workers related to mRNA vaccines due to perceived safety concerns: “The other thing is that vaccination, they say the priority is the health care workers, right, and I am sure most of the health care workers have not been vaccinated. Even myself, to be honest, I have not been vaccinated because of the issues we have with the vaccine, you know the rumors that it causes the clotting of the blood. Two of my colleagues died after receiving the vaccination—all these have been hearsay that people are hearing and so people are scared. So, nobody came out clearly and told us this vaccine is safe…and then the funny thing is when one is immunized nobody does follow up on the side effects; most of the people are scared of that or about that…So, I have changed my mind set to get the vaccine, but I am going to wait for the Johnson & Johnson. I do not want to get two doses—they said it is one jab and that is it. But I do not know when it is coming.” *(Female healthcare provider 2, rural, Garissa, Kenya).*

Given that generally, pregnant women were not included in COVID-19 vaccine clinical trials, stakeholders in both countries changed recommendations over the course of the pandemic related to which vaccines should be recommended to pregnant women. For example, this policymaker in Bangladesh indicated how the country first recommended OAZ over mRNA vaccines due to safety, but then changed recommendations and recommended OAZ and Pfizer vaccines, only recommending the Moderna vaccine if OAZ was not available. “Several vaccines are given to pregnant and breastfeeding women. We did not encourage them to get the Moderna vaccine, we provided another one namely AstraZeneca—Pfizer was also given. We are giving AstraZeneca now. We did not encourage the Moderna vaccine in the initial stage; later we observed that AstraZeneca, Pfizer all are good. We provided Moderna when those others were unavailable in stock.” *(National policymaker, urban, Dhaka, Bangladesh).* Similarly, this Bangladeshi policymaker commented on the fact that while the Pfizer vaccine was recommended for pregnant and lactating women, there were concerns about its side effects: “At the initial stage, according to the instruction of WHO, when pregnant and lactating women were suggested to have the Pfizer vaccine, we were not getting proper response. Even doctors also had fear about it. Because it was unknown—the long-term effect of the vaccine…Pfizer was given to pregnant and lactating women (based on the) instruction from WHO. It was said that Pfizer is less immunogenic/suitable for lactating mothers as well as pregnant women…We try to give vaccines as soon as possible to pregnant and lactating mother in our vaccine center. And we gave the most prestigious vaccine–Pfizer—to them…. Maybe the vaccines first introduced were not suitable (for pregnant women) according to their research findings. After getting research findings, they decided that Pfizer could be appropriate for pregnant women…If we wish to take Moderna, we are not able to take it!” *(District policymaker, urban, Gaibandha, Bangladesh).*

### 3.3 Preference for non-mRNA vaccines: health system compatibility and availability

In both countries, stakeholders alluded to changing recommendations related to vaccines given to pregnant and breastfeeding women. One key issue that drove changing recommendations was health system capacity as this Kenyan policymaker informed us that the country was giving OAZ because of its compatibility with the Kenyan health system: “We have been mainly been giving AstraZeneca; actually, most of the people we are talking of having been vaccinated have received this. It is more friendly to our system because it’s using all of the existing cold chain maintenance, but you find other vaccines, which are almost equally good. We seem to have other vaccines in our program, Johnson and Johnson, we have Moderna, we have Pfizer. As we improve our cold chain to handle those–they require temperatures that were not currently in our quoting system—we upgrade our infrastructure. I think a range of vaccines for COVID-19 also improved access because there is a range of vaccines available. People are free to make choices from variety and also improve their access.” *(National policymaker, urban, Nairobi, Kenya).* Availability also dictated preference as this Kenyan community leader alluded that there was a preference for the Johnson & Johnson vaccine due to a number of doses and also alluded that most community members were not aware of the vaccine they were getting: “I asked someone yesterday—someone who received the vaccination at Mama Lucy Hospital—the type of vaccine she received, and she said she never asked. She just went to receive the jab. So many do not ask…Somebody goes and just finds themselves vaccinated but they do not know what type it is…For now, AstraZeneca is what I have been hearing that people are getting.” *(Community member 2, urban, Nairobi, Kenya).*

## 4 Discussion

Factors leading to lack of confidence in COVID-19 vaccines for pregnant women were identified at the individual, health system, and environmental levels. Themes emerged related to the safety concerns for both mRNA and non-mRNA vaccines. Media reports influenced confidence for non-mRNA vaccines and respondents expressed a preference for a fewer number of doses with a non-mRNA vaccine. Policymakers expressed challenges for including mRNA vaccines within their current health systems that were not designed for their cold chain requirements. The initial unavailability of mRNA vaccines in many LMICs led to changing recommendations.

Both countries had gaps between COVID-19 vaccination policies in pregnancy and interpretations of policies by healthcare workers (14, 17). These results show that there is a lack of clarity among healthcare workers related not only to the overall recommendation itself but also to types of vaccine appropriate for use during pregnancy or while breastfeeding. A lack of information provided to healthcare workers or clear policies about maternal vaccines was found in several other studies in different countries, for both COVID-19 and other maternal vaccines (27, 28). As of 2022, WHO recommends vaccination for pregnant women and lists eight vaccines that can be used during pregnancy (29). These include both mRNA vaccines (Pfizer and Moderna), two viral vector vaccines (OAZ and Janssen/Johnson & Johnson), and two inactivated vaccines (Sinopharm and Sinovac).

At an early stage in the pandemic, acceptance of COVID-19 vaccines in LMICs was variable among the general population. In a systematic review of low-and lower-middle-income countries, Kenya had one of the highest rates of vaccine acceptance, more than 90%, while acceptance in Bangladesh was estimated at approximately 60% in a pooled analysis (30). Globally, COVID-19 vaccine acceptance among pregnant women was low. A systematic review of 15 studies found a pooled acceptance of 49.1% (95% CI, 42.3–56.0) with safety identified as a critical concern (31).

There have been global challenges to vaccine availability and access since early in the pandemic (32). Both Bangladesh and Kenya received only one vaccine product initially, in stark contrast to higher-income countries, which received multiple products for their initial vaccine rollouts. The contrast of initial vaccine product availability includes both monetary and structural factors (3, 20, 32). Policymakers in this study highlighted challenges to introducing mRNA vaccines into health systems; however, several countries, including both Kenya and Bangladesh, were able to adapt cold chain infrastructure to accommodate the lower temperature requirements. In Africa and Asia, the proportion of those vaccinated with mRNA vaccines is 22% (33).

This study found that mRNA technology itself was not a concern. However, unclear policies and recommendations, especially if there were multiple types of vaccines available, led to a lack of confidence in vaccines, even among healthcare workers and policymakers. A systematic review found mRNA COVID-19 vaccines to be safe and effective in pregnancy (34); these results should be used to improve confidence in mRNA vaccines among key stakeholders in maternal immunization. Including pregnant women in trials can be one way to improve confidence and has been identified by SAGE and regulators as a critical consideration as vaccine trials are planned (35). In addition, the mRNA platform holds promise for future vaccine development as several vaccine candidates across pathogens are leveraging mRNA technology.

There are limitations to this study. We conducted a qualitative study, and it was not designed to be generalizable. Given that we collected data in both countries during the height of the pandemic, participants likely felt pressure to have positive attitudes toward COVID-19 vaccines, and as such, social desirability bias is likely. The findings were heavily dependent on the cross-sectional nature of the study; policies related to COVID-19 vaccine eligibility were in flux in both countries during data collection. We also did not explicitly ask about vaccine preferences related to mRNA or non-mRNA COVID vaccines; data presented in the results are from participants that brought up vaccine preferences organically. Despite these limitations, this study has many strengths. It is one of the first that explored attitudes among a population at higher risk for severe COVID-19-related morbidity and mortality. As we did not ask explicitly about vaccine preferences, what emerged related to vaccine preferences is what organically arose when exploring the decision-making processes among these stakeholders. This study also provides insight related to how the decision-making process changes over time, within the context of changing policy recommendations and during a changing pandemic.

Clear vaccination policies, especially around which vaccines are preferred for use in pregnant women when multiple versions are available, could improve healthcare workers’ confidence. Not excluding pregnant women from trials of vaccines underdevelopment can provide the critical safety data that is needed to bolster the public’s confidence. mRNA vaccine technology holds promise for new vaccine development, and vaccine coverage will increase if the public’s confidence improves, reducing morbidity and mortality from vaccine-preventable diseases. Improving confidence will require increased transparency in clinical development and enhanced engagement of multiple stakeholders.

## Data Availability

The datasets presented in this article are not readily available because of ethical guidelines. Data will be shared upon reasonable request. Requests to access the datasets should be directed to RL, rlimaye@jhu.edu.
